# Manipulation of Auditory Inputs as Rehabilitation Therapy for Maladaptive Auditory Cortical Reorganization

**DOI:** 10.1155/2018/2546250

**Published:** 2018-05-20

**Authors:** Hidehiko Okamoto

**Affiliations:** Department of Physiology, School of Medicine, International University of Health and Welfare, 4-3 Kozunomori, Narita 286-8686, Japan

## Abstract

Neurophysiological and neuroimaging data suggest that the brains of not only children but also adults are reorganized based on sensory inputs and behaviors. Plastic changes in the brain are generally beneficial; however, maladaptive cortical reorganization in the auditory cortex may lead to hearing disorders such as tinnitus and hyperacusis. Recent studies attempted to noninvasively visualize pathological neural activity in the living human brain and reverse maladaptive cortical reorganization by the suitable manipulation of auditory inputs in order to alleviate detrimental auditory symptoms. The effects of the manipulation of auditory inputs on maladaptively reorganized brain were reviewed herein. The findings obtained indicate that rehabilitation therapy based on the manipulation of auditory inputs is an effective and safe approach for hearing disorders. The appropriate manipulation of sensory inputs guided by the visualization of pathological brain activities using recent neuroimaging techniques may contribute to the establishment of new clinical applications for affected individuals.

## 1. Introduction

Cortical structures in the adult brain were previously considered to be nonplastic; however, cortical reorganization appears to occur even in the adult brain. Previous animal studies [1, 2] demonstrated that auditory discrimination training induced frequency-specific receptive field plasticity in the adult guinea pig auditory cortex. Neural responses were maximally increased to the conditioned frequency and decreased to the pretraining best frequency and other frequencies. Robertson and Irvine [3] showed that restricted cochlear lesions resulted in auditory cortical frequency map reorganization. The area of the auditory cortex originally corresponding to the lesioned frequency range was partly occupied by an expanded representation of sound frequencies adjacent to the frequency range damaged by the lesion. These findings suggest that the manipulation of auditory inputs induces usage-dependent plasticity in the auditory cortex for the entire lifetime.

Advances in neuroimaging techniques that visualize living human brain activity have revealed that the human adult brain may also be reorganized based on sensory inputs from the surrounding environment and behaviors. Cortical reorganization generally occurs in a direction that is preferable for fulfilling demands. For example, the skills of professional musicians [4, 5] and jugglers [6, 7] have been attributed to reorganized cortical maps and connections in the human brain. Cortical reorganization generally contributes to an improved quality of life; however, it may also induce pathological phenomena [8]. Maladaptive cortical reorganization in the human brain has been suggested to play a major role in the emergence and maintenance of subjective detrimental symptoms such as phantom limb pain [9], focal hand dystonia [10], neuropathic pain [11], and tinnitus [12]. Therefore, the development of suitable behavioral rehabilitation approaches based on the manipulation of sensory inputs may be possible for the treatment of maladaptive cortical reorganization causing a deteriorated quality of life [8].

Maladaptive reorganization in the human auditory cortex was discussed herein, and neurorehabilitation approaches for tinnitus [13–15] and sudden sensorineural hearing loss [16, 17], which are based on cortical plasticity in the auditory system induced by the manipulation of auditory inputs, were also described.

## 2. Cortical Reorganization of Tinnitus

Subjective tinnitus is a phantom auditory sensation without any external sound source [18–20]. It severely deteriorates the quality of life of 1–3% of the adult population in industrialized countries [21–23]. Many approaches, including pharmacological therapy [24, 25], acupuncture [26], brain stimulation [27, 28], cognitive-behavioral therapy [29], and sound therapy [13, 30–33], have been attempted in order to reduce the phantom auditory sensation; however, their efficiencies currently remain unclear and a standard treatment for tinnitus has not yet been developed [34]. In order to establish an evidence-based treatment approach for tinnitus, it is important to understand the neural mechanisms underlying the emergence and maintenance of tinnitus symptoms.

Human neuroimaging studies using positron emission tomography (PET) [35, 36], single-photon emission computed tomography (SPECT) [37, 38], functional magnetic resonance imaging (MRI) [39], and magnetoencephalography (MEG) [40, 41] have revealed that subcortical and cortical plasticity play an important role in the perception of tinnitus. The modulation of neural activity in tinnitus patients appears to be related to hearing loss; however, previous studies suggested that subjective tinnitus is not always accompanied by hearing loss (for a review, see [42]). Most studies have investigated the auditory neural activity of humans or animals with hearing loss; therefore, it currently remains unclear whether the findings obtained in these studies reflect hearing loss, subjective tinnitus, or a combination of the two [43].

**Figure 1:**
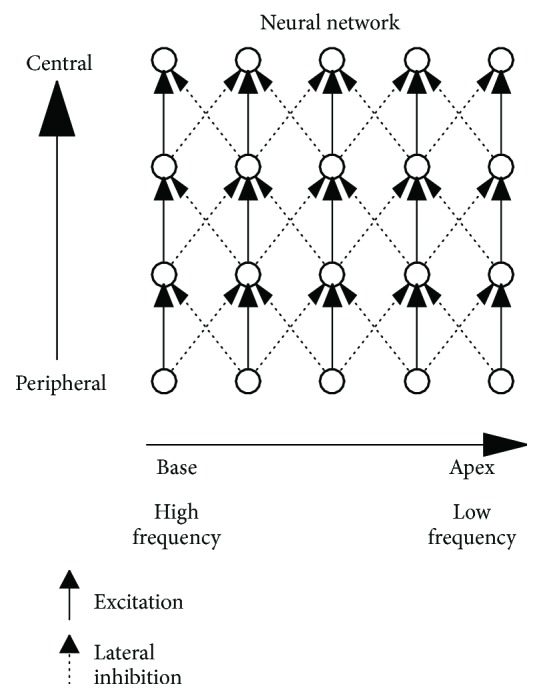
Schematically simplified excitatory and inhibitory neural networks from the peripheral to central auditory systems. Solid and dotted arrows indicate excitatory and inhibitory neural inputs, respectively (modified from Okamoto et al. [82]).

**Figure 2:**
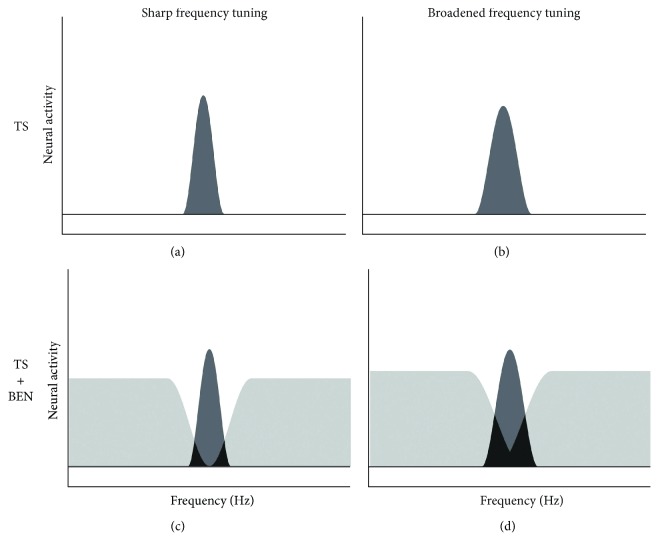
Schematic figures of neural activity elicited by a test stimulus (TS) and band-eliminated noise (BEN). Dark and light gray areas represent neural activities elicited solely by TS and solely by BEN, respectively. Black areas represent overlapped neural activities that can be activated by both TS and BEN. Since the black areas had already been activated by continuous BEN when TS was presented, the dark gray areas represent the neural activity elicited by the onset of TS. Left graphs (a and c) show sharp frequency tuning as indicated by narrow frequency distributions and right graphs (b and d) show broadened frequency tuning as indicated by rather wide frequency distributions. The overlapped areas (black areas) were more pronounced in broadened frequency tuning than in sharp frequency tuning (modified from [47]).

Auditory neural networks are composed of not only excitatory neurons but also inhibitory neurons, which contribute to sharpening frequency tuning by suppressing the excitatory activity of neighboring neurons (Figure 1) [44–46]. We hypothesized that damage to inhibitory neural networks does not necessarily worsen the hearing threshold, but may broaden frequency tuning in the auditory system. Therefore, we attempted to objectively measure population-level frequency tuning in unilateral tinnitus patients with similar hearing levels between their ears [47]. Previous studies [46, 48, 49] succeeded in objectively measuring population-level frequency tuning in the human auditory cortex. They measured auditory evoked N1m responses, which are the prominent auditory evoked component with a latency of approximately 100 msec [50], elicited by tonal test stimuli (TS) presented alone or together with a band-eliminated noise (BEN), in which a certain spectral frequency band centered at the TS frequency was eliminated from broadband noise. The neural activity elicited by TS or BEN partially overlap, and the degree of overlap depends on the sharpness of frequency tuning (Figure 2). Sharp frequency tuning results in the sparser overlap of neural activity than broadened frequency tuning, leading to larger neural activity elicited by the TS.

**Figure 3:**
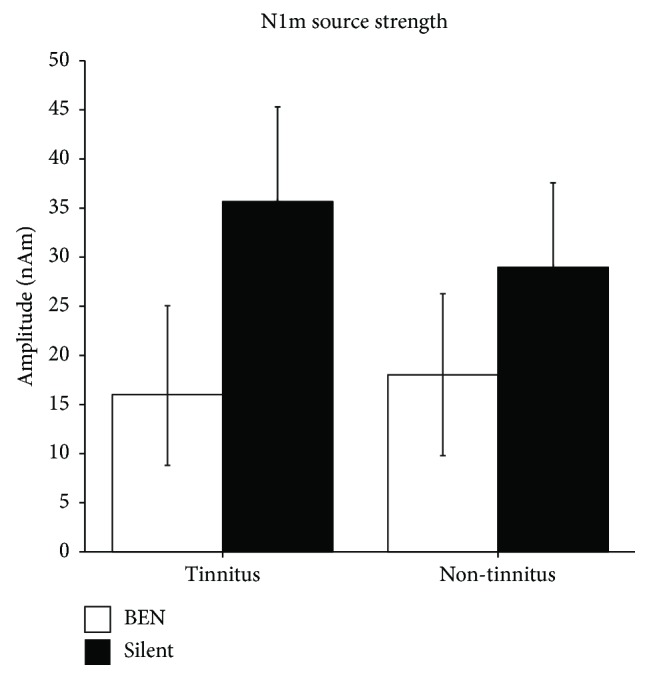
Group means of N1m source strengths elicited in tinnitus (left) and non-tinnitus (right) ears. Open and filled bars denote the “Band-eliminated noise (BEN)” and “Silent” conditions, respectively (adopted from [47]).

Our previous study [47] used a pure tone with each participant's tinnitus frequency as TS and presented them either in isolation or embedded in BEN, in which a frequency band around the tinnitus frequency was eliminated. The source strengths of N1m responses elicited by TS in each ear (“tinnitus ear” or “non-tinnitus ear”) and noise (“silent” and “BEN”) condition are presented with 95% confidence intervals in Figure 3. The N1m source strengths in silence were larger when TS were delivered to a tinnitus ear than to a non-tinnitus ear, whereas N1m source strengths elicited by TS in BEN were larger when TS were delivered to a non-tinnitus ear than to a tinnitus ear. Paired *t*-test indicated that the ratios of the N1m source strengths elicited in the BEN condition compared with those elicited in the silent condition were significantly greater for “non-tinnitus ear” than for “tinnitus ear” (*t*(6) = 3.16, *p* = 0.02, *d* = 1.20). As shown in Figure 2, neural activity elicited by TS and BEN can be classified into three groups: (1) neural activity evoked by TS (dark gray area), (2) neural activity evoked solely by BEN (light gray area), and (3) neural activity that can be elicited by both TS and BEN (black area). Group 3 (black area) represents the N1m source strength differences between the “BEN” and “silent” conditions because group 3 had already been activated by preceding BEN when TS was presented. The results in Figure 3 indicated that the BEN presented to a tinnitus ear caused a larger number of overlapped neural populations due to broadened frequency tuning (black area in Figure 2(d)) than that presented to a non-tinnitus ear (black area in Figure 2(c)). Since participants in this study had similar hearing levels between their ears, the findings obtained were related to the existence of subjective tinnitus, and not to hearing loss. Pathological alterations in inhibitory neural networks in the central auditory pathway appear to play an important role in the emergence and maintenance of subjective tinnitus symptoms [51–53].

## 3. Sound Therapy for Tinnitus

### 3.1. Tinnitus Retraining Therapy

The manipulation of sound inputs is one of the therapeutic approaches employed for tinnitus. Tinnitus retraining therapy (TRT) is one of the most widely used auditory stimulation approaches for tinnitus [54, 55]. TRT is mainly composed of two components: directive psychological counseling that aims at suppressing unpleasant tinnitus to a neutral signal and a noise generator that partially masks the tinnitus sound and reduces tinnitus-related neuronal activity. The combination of counseling and a noise generator appears to help tinnitus patients to reclassify the perception of tinnitus as a harmless sound signal [56, 57]. On the other hand, previous studies observed no significant additive effect because of the noise generators used in TRT [58–60]. Well-organized randomized clinical trials are needed in order to conclude the efficacy of TRT [61].

### 3.2. Tailor-Made Notched Music Training

It is reasonable to assume that appreciated music catches a listener's attention and more clearly affects his or her brain functions than the broadband noise widely used in TRT. The enjoyment of music plays an important role in activating the reward system of the brain and leads to effective reorganization in the human auditory cortex [62, 63]. A previous study [64] demonstrated that listening to notched music, in which a certain frequency band was eliminated, may reduce cortical activity corresponding to the center frequency of the eliminated frequency region. The decrement in neural activity may be caused by the aforementioned lateral inhibitory system (Figure 1). This finding prompted us to attempt to establish tailor-made notched music training (TMNMT) based on neurophysiological findings linking the manipulation of auditory inputs with cortical plasticity in the auditory system [13, 14, 65].

**Figure 4:**
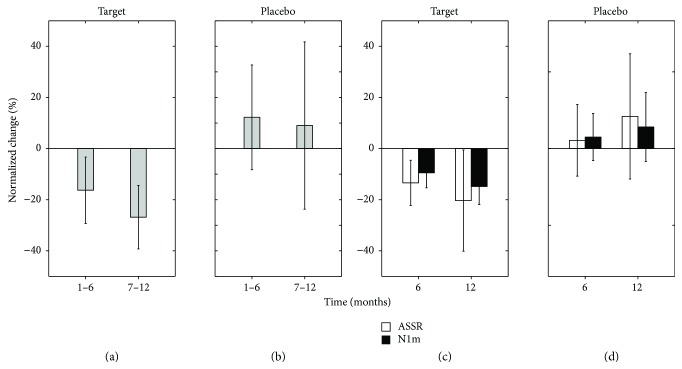
Normalized subjective tinnitus loudness (a, b) and normalized N1m and auditory steady state responses (ASSR) (c, d) 6 and 12 months after starting tailor-made notched music training (TMNMT) or placebo training. Error bars denote 95% confidence intervals (modified from [13]).

Tinnitus patients were assigned to one of two groups. Participants in the “target” group performed TMNMT; they listened to notched music that contained a constant frequency notch of one octave width centered at the individual tinnitus frequency. Participants in the “placebo” group listened to notched music that contained one octave moving notch sparing the tinnitus frequency octave. Over the course of the study, we recorded subjective tinnitus loudness and auditory-evoked responses elicited by each participant's tinnitus frequency TS.  Figure 4 shows behavioral and MEG findings, namely, the N1m response originating in the auditory belt area and the auditory steady-state response (ASSR) originating in the primary auditory cortex [66]. In participants who had received TMNMT, subjective tinnitus loudness, ASSR, and N1m were significantly lower than those measured before starting TMNMT. In contrast, in the placebo group, significant differences from the baseline were not observed in behavioral or MEG measurements.

We demonstrated that pathological alterations in the inhibitory system appeared to occur in the auditory system of tinnitus patients in the aforementioned study [47]. TMNMT may have decreased neural activity corresponding to the tinnitus frequency via lateral inhibitory neural connections, leading to reduced subjective tinnitus loudness. TMNMT is based on neurophysiological findings, and its target is the adaptive reorganization of the specific cortical area generating pathological tinnitus symptoms. In amendment to widely used indirect psychological treatment strategies, these findings introduce a different causal approach based on the manipulation of auditory inputs.

### 3.3. Hearing Aids for Tinnitus

Hearing aids are devices that amplify sound signals in order to compensate for hearing loss. It is widely known that the perception of tinnitus is often accompanied by sensorineural hearing loss. Therefore, the neural activity elicited by auditory signals amplified by the hearing aid may induce preferable reorganization in the auditory cortex and reduce the perception of tinnitus. However, clinical evidence to show that hearing aids suppress the perception of tinnitus is still limited [30]. Since the main aim of a hearing aid is to amplify sound signals related to speech perception, normal hearing aid devices are limited in their high-frequency output with an upper cut-off frequency of approximately 6 kHz. An acoustic stimulation with hearing aids was previously shown to be more effective for patients with a lower tinnitus pitch (<6 kHz) than a higher tinnitus pitch (>6 kHz) [67]. The amplification of a high frequency range is currently difficult due to technical reasons; however, the optimal settings of hearing aids that amplify the tinnitus frequency range instead of speech frequency range may effectively suppress the tinnitus perception in the future.

## 4. Constraint-Induced Sound Therapy against Sudden Sensorineural Hearing Loss

Sudden sensorineural hearing loss (SSHL) is an idiopathic condition characterized by the rapid loss of hearing without a clear pathological cause and is often accompanied by tinnitus symptoms [68]. However, limited information is currently available on SSHL, and it remains unclear whether standard corticosteroid therapy (SCT) is effective [69–73]. Previous studies [74–76] revealed that a sound stimulation of the healthy ear of unilateral hearing loss patients elicited similar neural activity in the ipsilateral and contralateral hemispheres, whereas a monaural stimulation caused neural activity to be strongly lateralized to the contralateral hemisphere in healthy subjects. Moreover, a previous study [77] demonstrated that the degree of plastic changes in hemispheric asymmetry induced by SSHL negatively correlated with hearing recovery.

**Figure 5:**
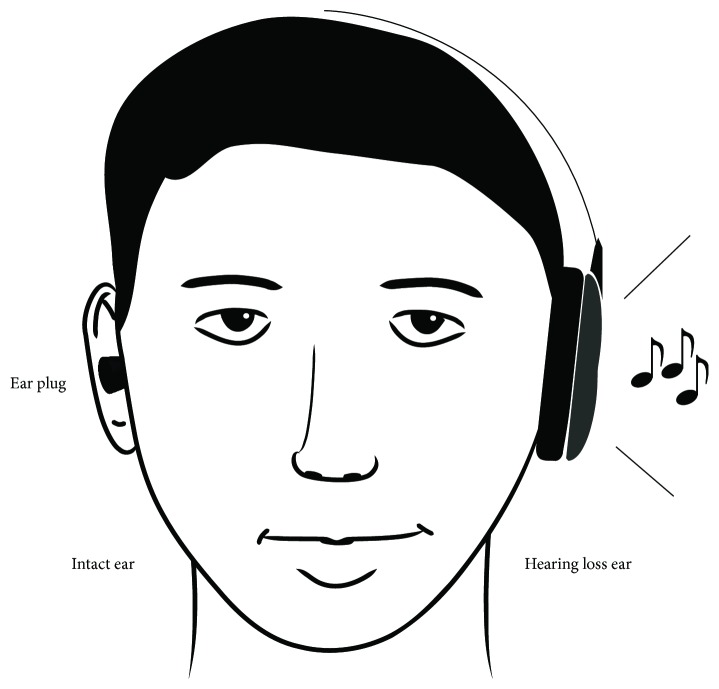
Schematic illustration of constraint-induced sound therapy (CIST). The intact ear was plugged in order to promote the usage of the hearing loss ear. Music was presented to the hearing loss ear (modified from [16]: drawing courtesy of Lothar Lagemann).

We focused on this maladaptive cortical reorganization initiated by SSHL and assumed that the prevention of plastic changes associated with SSHL is beneficial for hearing recovery [16, 17]. In an animal study [78], after loud noise exposure, cats housed in an enriched acoustic environment showed better-preserved tonotopic maps in the primary auditory cortex than those housed in a quiet environment. We applied this enriched acoustic environment together with the concept of “constraint-induced movement therapy” [79–81] to treat SSHL by preventing maladaptive reorganization in the human auditory cortex (constraint-induced sound therapy (CIST)). Constraint-induced movement therapy urges hemiparesis patients to use their affected limbs by prohibiting the usage of the healthy limbs with physical constraints, leading to the facilitation of neural recovery corresponding to the affected limbs. In our SSHL study, we plugged the outer ear canal of the healthy ear of SSHL patients (“constraint”) and forced them to use the hearing loss ear (Figure 5). Moreover, we asked them to listen to music (“enriched acoustic environment”) via their hearing loss ear for 6 hours per day during their hospitalization.

**Figure 6:**
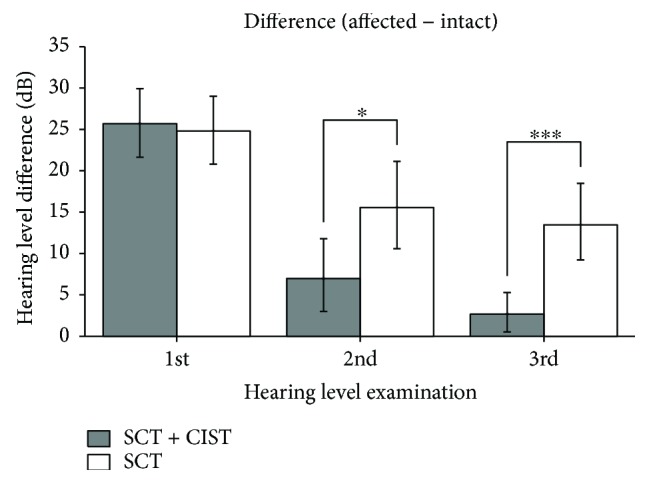
Mean hearing threshold differences between hearing loss and intact ears in the 1st (left: entering hospital), 2nd (center: leaving hospital), and 3rd (right: first visit as an outpatient after discharge) examinations. The gray and white bars indicate the group that received standard corticosteroid therapy (SCT) and constraint-induced sound therapy (CIST) and SCT alone, respectively. Error bars denote 95% confidence intervals (^∗^*p* < 0.05; ^∗∗∗^*p* < 0.001) (adopted from [16]).

Treatment outcomes were evaluated by comparing the hearing threshold levels of the two SSHL groups. One group was the “control” group that received SCT, while the other group was the “target” group that received CIST in combination with SCT. The mean threshold differences between the hearing loss and healthy ears were not significantly different between groups before starting the treatments (Figure 6 left: the 1st audiometric examination; *d* = 0.086). However, they differed in the 2nd (Figure 6 center: leaving hospital; *d* = 0.642) and 3rd (Figure 6 right: after discharge; *d* = 1.051) examinations. Participants in the “target” group recovered significantly better than those in the “placebo” group.

**Figure 7:**
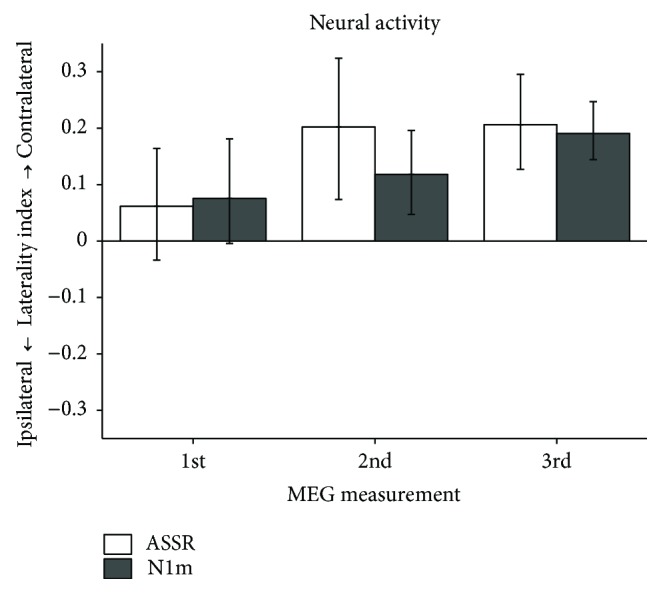
Laterality indices (LIs) of the auditory steady-state (ASSR: white bars) and N1m (gray bars) responses of participants who received constraint-induced sound therapy (CIST) in the 1st (left: entering hospital), 2nd (center: discharged from hospital), and 3rd (right: three months after being discharged) examinations. Error bars denote 95% confidence intervals (adopted from [16]).

Moreover, we measured the neural responses elicited by a monaural sound stimulation in SSHL patients in the “target” group. In order to assess the degree of plastic changes in hemispheric asymmetry [74–77], we calculated the laterality indices (LIs) of neural activities in the ipsilateral and contralateral hemispheres. LI was calculated as follows: (A − B)/(A + B), A = source strength elicited in the contralateral hemisphere, B = source strength elicited in the ipsilateral hemisphere.  Figure 7 shows the LIs of ASSR and N1m responses in the “target” group. In the 1st MEG recording (before the treatment), the LIs of ASSR and N1m were not significantly different from 0 (ASSR: *d* = 0.46; N1m: *d* = 0.61). This finding indicated that cortical plastic changes occurred in the auditory cortex of SSHL patients, as reported previously [74–77]. However, after CIST, the LIs of ASSR and N1m became more positive in the 2nd (ASSR: *d* = 1.16; N1m: *d* = 1.17) and 3rd examinations (ASSR: *d* = 1.78; N1m: *d* = 2.68), similar to normal-hearing individuals. CIST extensively activated auditory neurons corresponding to the affected ear, while neural activity corresponding to the intact ear was reduced due to the ear plug. CIST appeared to promote the recovery of neural activity related to the hearing loss ear and disturb the progression of maladaptive cortical reorganization. Therefore, the addition of safe and cost-effective CIST to routine corticosteroid treatments appears to be beneficial for SSHL patients.

## 5. Conclusion

Previous neurophysiological studies revealed that the auditory cortex may be reorganized based on auditory inputs, even in the adult brain. The utilization of previous findings on cortical plasticity induced by the manipulation of sensory inputs for the development of neurorehabilitation approaches appears to be effective for reducing or preventing maladaptive cortical plasticity and improving the quality of life of individuals with tinnitus [13, 14] and SSHL [16, 17]. Individual brain activity recorded by neuroimaging techniques may provide a key to optimizing the effectiveness of rehabilitation therapy for each patient. The combination of the noninvasive visualization of individual neural activity and a tailor-made neurorehabilitation approach adopting the manipulation of sensory inputs may rapidly increase in importance in the near future.
